# Influence of Environmental Factors on Biotic Responses to Nutrient Enrichment in Agricultural Streams^1^

**DOI:** 10.1111/j.1752-1688.2010.00430.x

**Published:** 2010-06

**Authors:** Terry R Maret, Christopher P Konrad, Andrew W Tranmer

**Keywords:** nutrients, agriculture, algae, macrophytes, eutrophication, monitoring

## Abstract

The influence of environmental factors on biotic responses to nutrients was examined in three diverse agricultural regions of the United States. Seventy wadeable sites were selected along an agricultural land use gradient while minimizing natural variation within each region. Nutrients, habitat, algae, macroinvertebrates, and macrophyte cover were sampled during a single summer low-flow period in 2006 or 2007. Continuous stream stage and water temperature were collected at each site for 30 days prior to sampling. Wide ranges of concentrations were found for total nitrogen (TN) (0.07-9.61 mg/l) and total phosphorus (TP) (<0.004-0.361 mg/l), but biotic responses including periphytic and sestonic chlorophyll *a* (RCHL and SCHL, respectively), and percent of stream bed with aquatic macrophyte (AQM) growth were not strongly related to concentrations of TN or TP. Pearson’s coefficient of determination (*R*^2^) for nutrients and biotic measures across all sites ranged from 0.08 to 0.32 and generally were not higher within each region. The biotic measures (RCHL, SCHL, and AQM) were combined in an index to evaluate eutrophic status across sites that could have different biotic responses to nutrient enrichment. Stepwise multiple regression identified TN, percent canopy, median riffle depth, and daily percent change in stage as significant factors for the eutrophic index (*R*^2^ = 0.50, *p* < 0.001). A TN threshold of 0.48 mg/l was identified where eutrophic index scores became less responsive to increasing TN concentrations, for all sites. Multiple plant growth indicators should be used when evaluating eutrophication, especially when streams contain an abundance of macrophytes.

## Introduction

Nutrient enrichment from anthropogenic sources has long been recognized as a significant impairment to aquatic ecosystems. Agricultural lands are commonly associated with high loading of non-point source nutrients to surface waters. The United States (U.S.) Geological Survey’s (USGS) National Water Quality Assessment (NAWQA) Program reported that streams draining agricultural watersheds transported high concentrations of phosphorus and nitrogen (Mueller and Spahr, 2006). The U.S. Environmental Protection Agency’s (USEPA) nationwide assessment on wadeable streams determined nitrogen and phosphorus concentrations were elevated above reference (or least disturbed) sites in 53 and 47% of the streams studied, respectively (USEPA, 2006).

Nutrient enrichment of streams from man-made sources can often cause eutrophication (i.e., increased primary productivity), which may result in excessive plant growth followed by severe diurnal variations in dissolved oxygen, reduction in available instream habitat, and a decrease in overall value for human uses (Welch, 1980; Dodds and Welch, 2000; Dodds *et al.*, 2009). These eutrophic factors can lead to reductions in aquatic biodiversity that favors invasive species over native species (Wang *et al.*, 2007; Maret *et al.*, 2008). These concerns combined with the difficulties in effectively controlling nutrients have led efforts by the USEPA to develop regional nutrient criteria using an ecoregional framework to help prevent nuisance growth of algae in streams (USEPA, 2000a). Two common biotic indicators of stream nutrient enrichment include chlorophyll *a* in periphyton (RCHL) and sestonic (SCHL) algae. Percent coverage of stream bottoms by aquatic macrophytes (AQM) including aquatic angiosperms, bryophytes, and filamentous benthic algae (Allan, 1995) has received much less attention (Hering *et al.*, 2006), even though excessive macrophyte growth can have major effects on beneficial uses of streams (Carpenter and Lodge, 1986; Suplee *et al.*, 2009).

Numerous studies have evaluated the influence of nutrients in experimental and natural stream channels and the concentrations or thresholds that stimulate plant growth (reviewed by Borchardt, 1996). Empirical regression models that predict algal biomass as a function of nutrient concentrations are often used to establish nutrient concentrations to protect streams (Lohman *et al.*, 1992; Dodds *et al.*, 2002; Stevenson *et al.*, 2006). However, the way nutrients interact with physical controls in regulating algal biomass in streams is not well understood. Nutrient concentrations often explain only a small to moderate amount of the variation in algal biomass (Munn *et al.*, 2010) and complex interactions of environmental factors including turbidity, temperature, light, substrate, grazer intensity, and hydrologic regime can impact accrual or loss of algal biomass (Lamberti and Resh, 1983; Munn *et al.*, 1989; Biggs, 1995, 1996a; Figueroa-Nieves *et al.*, 2006). Munn *et al.* (2010) determined the inclusion of the habitat variables temperature, canopy, velocity, slope, and base-flow index with nutrient concentrations improved the prediction of algal biomass in agricultural streams. An uneven spatial distribution of algae on a stream bed and variation in growth and biomass accrual over time may contribute to poor correspondence between algal biomass and nutrients in field studies (Morin and Cattaneo, 1992; Stevenson *et al.*, 2006), but biomass and nutrient concentrations can also vary diurnally and with daily or seasonal weather-related hydrologic events (Perkins and Jones, 1994; Figueroa-Nieves *et al.*, 2006).

Understanding how algal biomass responds to streamflow, habitat, and nutrient enrichment is critical in the development of effective management strategies. Thus, it is important to examine the direct factors among different landscapes responsible for influencing stream eutrophication. For example, Munn *et al.* (2009) found local factors (i.e., substrate and water temperature) were more important in controlling macroinvertebrates than landscape features such as ecoregions. Some studies that include sites with a gradient of nutrient concentrations have been effective at identifying factors responsible for eutrophication (Lohman *et al.*, 1992; Munn *et al.*, 2002; Porter *et al.*, 2008) and correlative or regression responses that model algal biomass and biotic responses to environmental factors can improve understanding of algal-nutrient relations in streams (Porter *et al.*, 2008).

Streamflow is an important variable that limits flora and fauna and strongly influences the functional status of stream ecosystems (Poff, 1997) and may explain in part why strong nutrient-biomass relations have not been found in many stream studies (Lohman *et al.*, 1992). Biggs and Close (1989) recommend that algal biomass data always be viewed within the context of antecedent streamflow conditions. High streamflow events can dislodge algae or can be beneficial to algal communities if grazing herbivores are reduced (Koetsier, 2005) whereas low streamflows can cause desiccation of algal communities (Stanley *et al.*, 1997).

Flow regimes also directly influence the availability of nutrients and dissolved oxygen for algal growth and can influence water temperature and turbidity that directly affects water clarity. However, continuously measured streamflow data are often unavailable for many stream locations, and can be expensive to collect. However, McMahon *et al.* (2003) showed that for certain hydrologic aspects (e.g., measurements of duration and relative change in flow), stage data were as useful as streamflow based metrics. Thus, this study investigated if stage data could characterize hydrologic conditions prior to sampling to better evaluate nutrient-algal growth as it relates to development of nutrient criteria.

The objectives of this study were to: (1) compare the responses of aquatic plant growth (i.e., RCHL biomass, SCHL concentration, and percent AQM) to nutrient concentrations among streams in three diverse agricultural regions of the U.S.; (2) evaluate whether proximate environmental factors influence biological responses to nutrients; and (3) examine changes in biotic responses along nutrient gradients for thresholds that may lead to nuisance plant growth. Understanding the influence of streamflow and habitat on nutrient-algal relationships among different ecoregions would help establish the range of expected conditions and the factors that regulate those ranges. USEPA (2000a) recommends using information about habitat and streamflow conditions, but offers no specific guidelines on integrating these variables into nutrient criteria development. This paper strengthens current approaches to assess stream eutrophication and to develop nutrient criteria for major agricultural regions of the U.S.

## Methods

### Study Area Description

**FIGURE 1:**
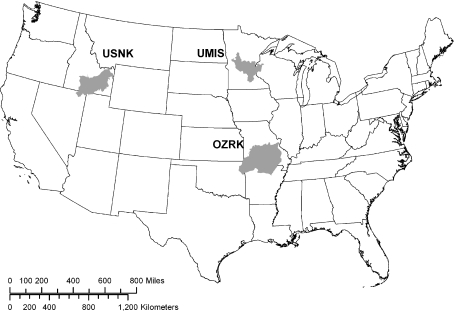
Location of the Streams Sampled Within the Ozark Highlands (OZRK), Upper Mississippi (UMIS), and Upper Snake (USNK) Regions (gray-shaded areas) Sampled by U.S. Geological Survey, 2006-2007. See Appendix 1 for names and locations of sample sites.

This study was conducted in three regions (or study areas) characterized by a gradient of agricultural land use practices ranging from minimal to intense (Figure 1, Table 1). These included: the Ozark Highlands (OZRK) in Arkansas, Missouri, and Oklahoma; Upper Mississippi (UMIS) in Minnesota and Wisconsin; and Upper Snake River (USNK) in Idaho and Nevada. Field activities were conducted in 2006 at the OZRK, and in 2007 at the UMIS and USNK. The USEPA (2000b) has developed ecoregional nutrient criteria that were intended as starting points for states and tribes to develop more refined and locally relevant nutrient criteria. For the three study areas in this study, total nitrogen (TN) and total phosphorus (TP) ecoregional criteria range from 0.31 to 0.54 mg/l and 0.010 to 0.033 mg/l, respectively (Table 1).

**TABLE 1:** Summary of Dominant Physical Characteristics (values are means with ranges) and USEPA (2000b) Ecoregional Nutrient Criteria.

Parameter	OZRK (*n* = 22)	UMIS (*n* = 18)	USNK (*n* = 30)
Ecoregion level III	Ozark Highlands	North Central Hardwood Forest	Snake River Plain Northern Basin and Range
Aggregate nutrient ecoregion	Central and Eastern Forested Uplands	Glaciated Dairy Region	Xeric West
Climate	Temperate Highlands	Humid Plains	Arid Intermontane
Site elevation (m)	258 (119-388)	320 (213-432)	1,315 (776-1,914)
Basin size (km^2^)	142 (75-255)	249 (31-634)	815 (0.22-5,225)
Agricultural land (%)	39.4 (0.5-86.9)	48.5 (5.1-94.8)	25.7 (<0.1-95.9)
Precipitation (cm/year)	123 (118-130)	78 (69-88)	45 (22-75)
Ecoregional nutrient criteria			
Total nitrogen (mg/l)	0.31	0.54	0.38
Total phosphorus (mg/l)	0.01	0.033	0.022

Notes: Data summaries from U.S. Geological Survey Geographic Information (GIS) Sources. See Brightbill and Munn (2008) for a summary of methods used to derive GIS summaries. Ecoregion level III names from Omernik (1987); aggregate nutrient ecoregions from USEPA (2000a).

All three regions had significant agricultural lands ranging from an average of 26% in the USNK to 49% in the UMIS. Livestock farming is common among all three regions. Long-term average precipitation for the OZRK, UMIS, and USNK are about 123, 78, and 45 cm/year, respectively. The eastern regions UMIS and OZRK are in biomes with increased humidity with agriculture relying primarily on natural rainfall and smaller drainage basins (142-249 km^2^). The OZRK region is entirely within the Ozark Highlands aggregate ecoregion and the UMIS region lies within the Glaciated Dairy Region aggregate ecoregion (Table 1). The western region (USNK) is located in the Xeric West aggregate ecoregion, relies more on irrigation practices, has larger average drainage basins, and higher elevation. High streamflows in this region are associated with spring snowmelt rather than rainfall events. Descriptions for aggregate ecoregions are found at USEPA (2000b).

### Site Selection and Study Design

A combination of a geographic information system and field reconnaissance methods were used to select the 70 study sites. Site selection attempted to identify study sites within a single ecoregion having similar hydrologic landscape, soils, climate, land use, and biota (Omernik, 1987). Wadeable streams with a gradient of agricultural land use and range of nutrient conditions were selected. Independent basins were selected and the National Hydrologic Dataset used to verify the locations of streams (Brightbill and Munn, 2008). The smallest basins were indicative of large springs located in the USNK. Reconnaissance surveys were completed for each candidate stream to evaluate access, habitat conditions, and stream size. When existing information was not available, field measurements of nutrients, dissolved oxygen, pH, specific conductance, and temperature were measured. Field forms were completed that documented observations for habitat quality and flow characteristics. Basins containing USGS continuous gages were targeted if they met the desired conditions. Information on nutrient concentrations were most important in the site selection process to insure sites captured a gradient of nutrients found in each study area. This process resulted in the selection of 18 to 30 sites within each region that had complete datasets for analysis (Appendix S1). A more detailed description of the site selection process can be found in Brightbill and Munn (2008).

Biological and habitat assessments targeted the growing season (July-August) when streams reached stable summer low-flow conditions. The focus of this assessment was at the reach and microhabitat (riffle) scale using standard sampling (Table 2). A reach was defined as a repetition of a geomorphic sequence (e.g., two riffles and two pools), or 20 channel widths if repetitive units were not available and at least 150 m in length (Fitzpatrick *et al.*, 1998). Many of the instream habitat variables selected for analysis follow the conceptual template suggested by Biggs (1996b) to regulate aquatic plant growth in streams (e.g., nutrients, light, streamflow, substrate, temperature, and grazing by macroinvertebrates).

**TABLE 2:** Summary of Chemical, Physical, and Biological Variables Calculated or Measured for All Sites and Each Study Area, 2006-2007.

			Study Area		
Variable	Abbreviation	Units	OZRK (*n* = 22)	UMIS (*n* = 18)	USNK (*n* = 30)
Chemical					
Total nitrogen	TN	mg/l	0.07-4.71	0.46-9.61	0.11-3.91
Total phosphorus	TP	mg/l	<0.004-0.062	0.033-0.361	0.010-0.159
Conductivity	COND	μS/cm	268-529	252-1,089	82-1,560
Physical					
Turbidity	TURB	NTU	<0.1-8.0	<0.1-50.0	0.1-31.5
Canopy shading	CAN	%	12.0-69.7	10.2-83.7	6.2-70.1
Discharge	Q	m^3^/s	0.029-0.461	0.003-0.688	0.033-3.416
Gradient	GRAD	%	0.081-0.546	0.013-0.367	0.027-3.200
Daily change in stage	DSTAGE	%	0.1-8.6	0.4-3.8	<0.1-2.8
Base flow index	BFI	%	26.4-49.6	45.6-67.4	64.6-86.6
High flow index	HFI	Unitless	0.376-2.418	0.682-1.771	0.347-1.286
Dimensionless shear stress	DIMSHEAR	Unitless	0.000-0.030	0.000-0.020	0.000-0.040
Water temperature	TEMP	C	18.6-26.1	14.2-25.8	14.5-24.9
Median substrate^1^	SUBSTR	mm	1-96	9-384	9-384
Median depth^1^	DEPTH	m	0.067-0.213	0.030-0.274	0.08-0.50
Median velocity^1^	VEL	m/s	0.07-0.81	0.02-0.48	0.03-0.59
Biological					
Periphyton chlorophyll *a*^1^	RCHL	mg/m^2^	10.4-124.7	8.3-172.3	2.4-149.9
Seston chlorophyll *a*	SCHL	μg/l	0.2-2.1	0.5-36.4	0.1-6.0
Aquatic macrophytes	AQM	%	0.0-50.0	0.0-62.9	0.0-90.7
Invertebrate scrapers^1^	SCBIO	g/m^2^	0.1-25.5	<0.1-7.4	<0.1-17.7
Eutrophic index	EI	Unitless	6.3-28.3	5.9-44.7	1.2-37.6

1 Variable represents microhabitat (riffle) within reach.

### Water Chemistry

Nutrient samples were collected twice at each site with the first sample collected ca 30 days prior to the biological and the second sample collected concurrently with biological samples. A paired *t*-test found there was no significant difference (*p* > 0.05) in nutrient concentrations between time periods, so a mean nutrient concentration for each site was used in the analysis. Mean differences for paired TN and TP concentration (*n* = 70) were 0.74 and 0.022 mg/l, respectively. Nutrient samples were collected using a depth- and width-integrated sampling method or one to three mid-channel grab samples composited when flow velocities and/or water depths were too low to allow isokinetic sampling using field equipment (Shelton, 1994). All samples were placed on ice and shipped within 24 hours of collection to the USGS National Water Quality Laboratory (NWQL) in Arvada, Colorado, for analysis. Instantaneous field measurements included conductivity and discharge. Turbidity was measured with a Hach meter model 2100P (Hach, Loveland, Colorado). Nutrient samples were analyzed for TN and TP. Alkaline persulfate digestion was used to determine TN (Patton and Kryskalla, 2003) and microkjeldahl digestion was used for TP (Patton and Truitt, 2000). TN and TP indicate nutrient availability as well or better than dissolved parameters and are most commonly used in nutrient criteria development (Dodds and Welch, 2000). Method detection levels for TN and TP were 0.03 and 0.004 mg/l, respectively. Censored values were rare, with only one occurrence for TP, which was assigned a value of one-half the method detection level prior to analysis. More details about sampling or laboratory methods can be found in Brightbill and Munn (2008).

### Biological Samples

A Slack sampler (500 micron mesh, 0.25 m^2^ sample area, total area of 1.25 m^2^ per sample) was used to collect semi-quantitative invertebrate samples from five riffle locations throughout the reach. Sample processing involved elutriation to remove inorganic debris, compositing subsamples, and preservation with 10% formalin (Moulton *et al.*, 2002). Macroinvertebrates were sorted using standard 500 count procedures, identified to the lowest practical taxonomic level, and biomass (g/m^2^) determined (Moulton *et al.*, 2000). Biomass estimated were determined by drying at 60°C to a constant weight. Large-rare taxa (e.g., crayfish) were not included in the biomass determinations. The biomass of scrapers was determined based on the ratio of macroinvertebrates that were scrapers. Trophic groupings were assigned using the USGS Invertebrate Data Analysis System (Cuffney, 2003).

Chlorophyll *a* samples were collected from coarse substrate (gravel or larger) and the water column. For RCHL samples, five rocks were collected in close proximity to each of the invertebrate samples and the algae were scraped from a consistent area on each rock and 25 subsamples composited. Water column samples for SCHL were collected by methods used for nutrient sampling. RCHL and SCHL samples were filtered onto a Whatman 47-mm glass fiber filter and analyzed for chlorophyll *a* and pheophytin biomass (Moulton *et al.*, 2002). Algal biomass samples were shipped on dry ice to the NWQL for analysis. Chlorophyll α and pheophytin were determined using an adaptation of EPA method 445.0 (Arar and Colling, 1997), with a detection limit of 0.1 μg/l.

Visual estimates of percent AQM (including macroalgae with filaments >2 cm) coverage were made at five points across each of 11 equidistant transects along the study reach. A mean percentage for the 55 observations was calculated to represent the reach. Forty percent coverage of the stream bottom with AQM was selected to characterize eutrophic conditions with excessive plant growth. This was derived by taking the midpoint of 20-60% coverage reported by Suplee *et al.* (2009) as undesirable growth in Montana streams. Similarly, Chambers *et al.* (1999) reported levels of 10 to 50% may be considered nuisance growth in Canadian streams. We also identified a group of sites that appeared to have excessive AQM growth covering more than 40% of the stream bottom.

### Habitat

Physical habitat was assessed at the stream reach and microhabitat (riffle) scale. A total of 11 equidistant transects oriented perpendicular to streamflow were established throughout the reach, with channel width (m) measured at each transect. Mean percent canopy cover was measured using a densiometer with measurements made at the center channel and each bank edge along each transect. Measurements were averaged for each transect and then combined for an overall mean percent canopy cover for each reach. Reach gradient was determined using a surveyor’s level with gradient calculated at the water-surface. Characterization of riffle microhabitats consisted of measurements of near-bed velocity (ca 2 cm from stream bottom), depth, and substrate size at each location where algae and macroinvertebrates were sampled. A modified Wolman pebble count was completed at riffle habitats sampled (Wolman, 1954). This consisted of selecting and measuring substrate particles at each of five locations where macroinvertebrates and algae were sampled using a square PVC frame with 20 equally spaced locations around the frame. A total of 100 particles were measured to characterize the substrate size into 10 substrate size classes for the riffle habitats sampled. Median values of velocity, depth, and substrate were calculated to represent microhabitat measurements. Additional detail on methods used to collect habitat data can be found in Fitzpatrick *et al.* (1998).

### Continuous Stage and Water Temperature

Continuous stream-stage data and water temperature were collected at existing USGS gages (23 sites) or vented submersible pressure transducers (47 sites) with a 15-min to 1-hour time step. Stage and temperature data from sites within each region were processed to 1-hour intervals and used to derive daily statistics using a common period of record of 30 days prior to the biological sampling (McMahon *et al.*, 2003). This period of record has been suggested to be adequate for biomass accrual in streams (Cattaneo and Amireault, 1992; Lohman *et al.*, 1992; Snyder *et al.*, 2002) and it allows for maximum biomass accrual, but avoids subsequent sloughing.

Stage data, which have arbitrary datum, were adjusted using the mean bed elevation so that the daily values of stage provided a depth time series. Mean daily percent change in stage for the 30 days prior to the ecological assessment was calculated and normalized by median stage for this same 30-day period.

A surveyed channel cross-section at the location of the transducer was used to calculate cross-sectional area. Discharge was measured at the time of sampling at each site using methods described in Rantz *et al.* (1982) or using established rating curves at co-located USGS gages. Daily-stage time-series data for each site were inspected for continuity and extreme high- or low-flow events. Dimensionless shear stress (DIMSHEAR), which provides measure of the force of streamflow relative to the size of bed material, was calculated for each site to evaluate bed stability: 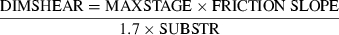
(1) where MAXSTAGE was the maximum daily stage for the period of record and FRICTION SLOPE was estimated from the vertical velocity gradient and bed substrate (SUBSTR). A threshold of DIMSHEAR > 0.045 was used to assess whether high flows were likely to have entrained bed material during the 30 days prior to sampling (Konrad *et al.*, 2002).

Streamflow records from long-term USGS gages were used to estimate annual maximum daily streamflow at each site for each of the 10 years prior to biological sampling (Dave Wolock, USGS, January 15, 2009, personal communication). A high-flow index, representing the magnitude of high flow during the year of sampling relative to median annual high flow, was calculated as maximum daily streamflow for the year of sampling at each site normalized by median annual maximum streamflow for the 10-year period. Base-flow index, the component of streamflow that can be attributed to groundwater discharge into streams, was estimated for each site from the national base-flow index 1-km resolution dataset developed by Wolock (2003).

### Data Analyses

All statistics were calculated using either Systat^©^ version 11.0 (Wilkinson, 2004) or R (R Development Core Team, 2005). Scatter plots were examined for outliers and spurious correlations. Correlation coefficients (Spearman’s rho) among variables were examined to identify and reduce redundancy. Variables with skewed distributions were log_10_ or square-root transformed to normalize their distributions. Following transformation of the data, ANOVA was used to determine if there were significant differences in variables among regions, followed by Tukey multiple comparisons

Bivariate linear regression models were developed to examine the influence of nutrients on eutrophic indicators (RCHL, SCHL, and AQM). Piecewise regression or segmented regression (Toms and Lesperance, 2003) was used to identify breakpoints or thresholds in biotic responses and nutrient concentrations. This approach to modeling data identifies regression changes at one or more points along the range of the independent variable. A LOWESS (LOcally WEighted Scatterplot Smoothing) technique, a robust nonparametric description of data patterns (Helsel and Hirsch, 2002), was initially used to examine the number and location of the breakpoints. The influence of habitat and biotic factors on nutrient-eutrophication response was assessed using multiple regression with the RCHL, SCHL, or AQM as the dependent variable and TN, TP, and selected habitat variables as the potential independent variables (see Table 2). Conductivity was evaluated as an explanatory variable as it has been shown to be a surrogate for nutrients (Biggs, 1995; Munn *et al.*, 2002).

An exhaustive stepwise search identified the multivariate models with the lowest Akaike’s information criterion (Helsel and Hirsch, 2002) where all independent variables had statistically significant coefficients (*p* < 0.05 that the coefficient = 0) and multiple colinearity was not an issue (variance inflation factor <2 for all explanatory variables). Three binary variables indicating regional membership were created to determine if region-specific models were warranted.

A eutrophic index (EI) was calculated using three metrics that reflect instream primary productivity (RCHL, SCHL, and AQM) to nutrients expressing eutrophication. Each metric was scaled to an expected low and high for all sites so the range of scoring approximated 0-100. This is similar to the scoring system outlined in Hughes *et al.* (1998): 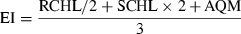
(2)

This empirical EI is not intended to be transferrable to other investigations because it is based on the observed range of biotic responses at our sites. Multiple linear regression, as described above, was used to develop a model expressing the influence of nutrients, habitat, and biotic factors.

## Results

### Physical and Chemical Characteristics

Daily-stage records from 30 days prior to biological sampling indicated no high flows capable of substantial bed material entrainment (DIMSHEAR < 0.045) or extreme low flows in reach habitat sampled. A few sites had high values (DIMSHEAR = 0.040), but were stable at this flow. At these sites, the reaches had boulder steps with very high shear stress. As a result, the reach average shear stress calculations were higher than and unrepresentative of shear stress at the location of bed material and biological sampling. Daily change in stage was generally low at the sites (median of about 1%), though it did range up to about 9% (Table 2). Mean high-flow index values for each region were near 1.0 indicating the year of sampling was generally similar to the long-term 10-year period prior to sampling. Mean base-flow index values among study regions were significantly different, with the USNK sites having the largest groundwater contributions to total streamflow (76%). There were no statistical differences among regions for conductivity, turbidity, and canopy shading.

**FIGURE 2:**
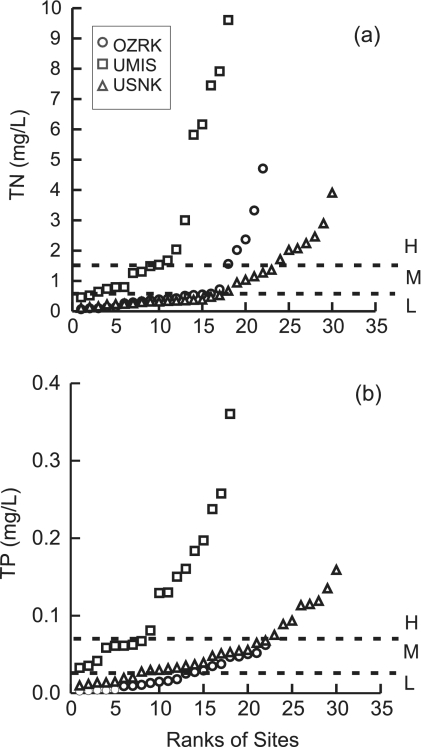
Cumulative Distributions of (a) Total Nitrogen (TN) and (b) Total Phosphorus (TP) Across All Study Sites, *n* = 70. Dashed lines indicate nutrient (TN and TP) trophic boundaries suggested by Dodds *et al.* (1998). L, low enrichment; M, moderate enrichment; H, high enrichment.

The sites had a gradient of TN and TP concentrations within and among regions (Figures 2a and 2b) and represented a wide trophic status from low- to high-nutrient conditions. The gradient and range for TP concentrations was the weakest among OZRK sites. Concentrations of TN ranged from 0.07 to 9.61 mg/l for all sites (Table 2). Concentrations of TP ranged from <0.004 to 0.361 mg/l, with the UMIS having significantly greater mean concentration (0.128 mg/l) and the OZRK significantly lower mean concentrations (0.024 mg/l) than other regions. There was a positive relation between TN and TP (*r* = 0.55, *p* < 0.05) (Table 3). Conductivity was also positively related to TN (*r* = 0.43, *p* < 0.05).

**TABLE 3:** Spearman’s Rho Correlation Coefficients Among Variables in Streams of the OZRK, UMIS, and USNK Study Areas (*n* = 70, bold denotes *p* < 0.05, with Bonferroni adjustment).

	TN	TP	GRAD	SUBSTR	CAN	TURB	DEPTH	VEL	Q	DIMSHEAR	TEMP	COND	DSTAGE	HFI	BFI	SCBIO	RCHL	AQM	SCHL
TP	**0.55**																		
GRAD	−0.22	−0.26																	
SUBSTR	−0.21	−0.41	**0.57**																
CAN	0.07	0.13	0.20	−0.01															
TURB	0.14	**0.54**	−0.14	−0.28	−0.09														
DEPTH	0.10	0.01	0.08	0.02	0.02	−0.07													
VEL	0.00	−0.21	0.09	0.16	0.21	−0.04	−0.15												
Q	0.05	−0.01	−0.02	0.01	−0.06	−0.01	**0.72**	0.02											
SHEAR	0.07	−0.09	0.11	0.00	0.05	0.03	−0.12	**0.75**	0.02										
TEMP	−0.11	−0.16	−0.31	−0.17	−0.21	−0.06	−0.28	0.20	−**0.40**	0.24									
COND	**0.43**	0.28	−0.10	−0.19	−0.12	0.18	−0.02	0.02	−0.05	0.14	0.01								
STAGE	−0.01	0.14	−0.11	−0.31	0.18	0.00	−0.08	−0.05	−0.21	0.14	0.31	0.01							
HFI	−0.07	−0.21	0.04	0.07	0.12	−0.09	−0.06	0.25	−0.05	0.28	0.27	0.02	0.05						
BFI	0.00	0.27	0.23	0.17	−0.09	0.32	0.32	−0.36	**0.40**	−0.29	−**0.68**	0.08	−0.29	−0.24					
SCBIO	0.10	−0.02	0.07	−0.05	0.09	−0.20	0.04	0.11	−0.02	0.08	0.09	−0.10	0.06	0.19	−0.16				
RCHL	0.22	0.02	−0.10	−0.02	−0.20	0.21	0.12	0.03	0.12	0.11	0.17	0.13	0.14	0.15	−0.05	0.11			
AQM	0.26	0.09	−0.04	0.02	−0.15	−0.18	**0.55**	−0.21	0.33	−0.12	−0.17	0.18	−0.02	−0.11	0.16	0.09	−0.11		
SCHL	0.38	**0.50**	−0.33	−**0.42**	−0.17	**0.53**	−0.16	−0.13	−0.10	−0.09	−0.05	0.20	0.06	−0.14	0.20	−0.15	0.14	−0.11	
EI	**0.53**	0.33	−0.22	−0.17	−0.30	0.06	0.29	−0.32	0.18	−0.09	−0.02	0.37	0.18	0.02	0.14	0.05	**0.54**	**0.52**	0.29

Note: See Table 2 for variable definitions.

**FIGURE 3:**
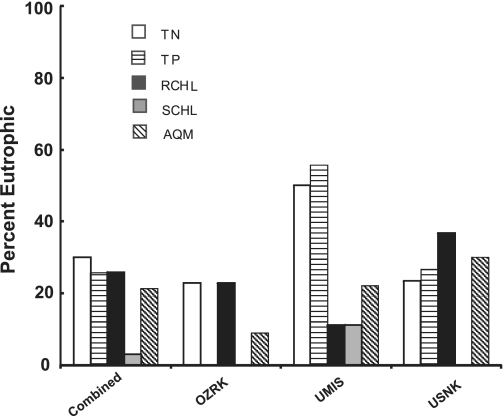
The Relative Percentage of All Sites and Individual Regions Classified as Eutrophic (highly enriched) on the Basis of Total Nitrogen (TN > 1.5 mg/l), Total Phosphorus (TP > 0.75), Periphytic Chlorophyll *a* (RCHL > 70 mg/m^2^), Seston Chlorophyll *a* (SCHL > 30 ug/l) (Dodds *et al.*, 1998), and Aquatic Macrophyte (AQM) Coverage of Stream Bottom >40% (Chambers *et al.*, 1999; Suplee *et al.*, 2009).

The classification scheme of Dodds *et al.* (1998) indicated that about 26-30% of all sites were highly enriched based on the TP and TN concentrations, respectively (Figure 3). The UMIS region had the highest percentage of enriched sites with 50 and 56% of the sites exceeding TP and TN concentrations, respectively. The OZRK region had only about 23% enriched sites based on TN concentrations and no sites exceeding the highly enriched boundary for TP.

### Biological Measures of Eutrophication

Biomass of RCHL ranged from 2.4 to 172.3 mg/m^2^ for all sites with no significant difference between regions (Table 2). The UMIS had the lowest mean RCHL biomass (46.6 mg/m^2^) among regions despite the highest mean TN and TP concentrations among regions. Concentration of SCHL ranged from 0.1 to 36.4 μg/l, with the UMIS mean (7.6 μg/l) significantly higher than the other regions. The AQM were highly variable and ranged from 0 to 91%, with no significant differences in regions. The highest visual percentages for AQM were found in the USNK region where *Potomogeton* sp. or *Cladophora* sp. often comprised the majority of the streambed coverage. A positive relation was found between AQM and median depth (*r* = 0.55, *p* < 0.05) (Table 3).

**FIGURE 4:**
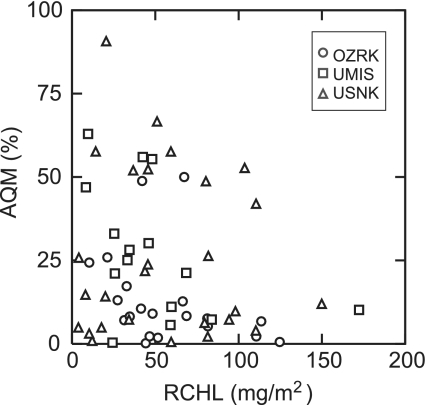
Relation of Periphytic Chlorophyll *a* (RCHL) to Macrophyte (AQM) Percent Coverage of Stream Bottom for All Study Sites (*n* = 70).

High values of AQM and RCHL were inversely related with sites forming a wedge-shaped scatterplot (Figure 4). There was a group of sites with AQM that approached or exceeded 40% bottom coverage within the study reach that indicated excessive plant growth. However, many of these same sites had relatively low (<50 mg/m^2^) RCHL biomass.

The invertebrate scraper biomass ranged from <0.1 to 26 g/m^2^ for all sites (Table 2). Molluscs exceeded 3 g/m^2^ at 16, 4, and 8 sites in the OZRK, UMIS, and USNK study units, respectively. The greatest mean values (5.7 g/m^2^) were found for the OZRK sites, followed by USNK (2.5 g/m^2^) and UMIS (1.0 g/m^2^). The largest contributor to the biomass in the OZRK was *Elimia potosiensis*, a native snail. The USNK sites with the greatest biomass were a result of *Potamopyrgus antipodarum*, an invasive snail. There were no statistically significant correlations between invertebrate scraper biomass and other environmental variables measured at study sites (Table 3).

For all sites combined, the RCHL measurements resulted in the most sites being classified as eutrophic (26%), followed by AQM (21%) and SCHL (3%, Figure 3). The USNK region had the highest number of sites (30%) that exceeded the AQM eutrophic boundary, followed by UMIS (22%) and OZRK (9%). The USNK also had the highest number of sites (37%) classified as eutrophic based on RCHL measurements, followed by OZRK (23%) and UMIS (11%). With the exception of the UMIS, other regions had no sites classified as eutrophic based on SCHL measurements.

### Nutrient-Biotic Response Models

**FIGURE 5:**
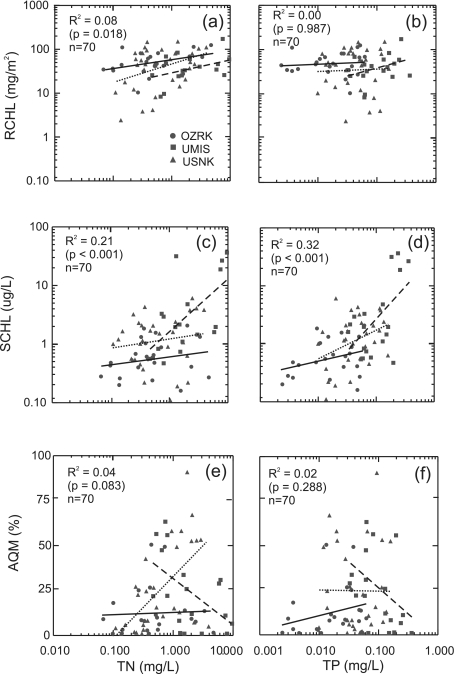
Bivariate Plots of the Biotic Response Variables Periphytic (a and b) Chlorophyll *a* (RCHL), (c and d) Sestonic Chlorophyll *a* (SCHL), and (e and f) Aquatic Macrophyte (AQM) Percent Coverage and Total Nitrogen (TN) and Total Phosphorus (TP) Concentrations. The lines indicate the best fit linear regression for streams in the OZRK (solid line), UMIS (dashed line), and USNK (dotted line). Regression equations of all sites combined and individual regions can be found in Table 4.

Variability in the three biotic responses to nutrient concentrations was high in all regions (Figure 5). Bivariate regression analyses on all sites and individual regions produced 9 out of 24 significant models among TN and TP and the three biotic response variables (Table 4). The amount of variation explained by these significant models was 8-32% (Figures 5a and 5d). TN was significant (*R*^2^ = 0.08, *p* < 0.018) in predicting RCHL for all sites (Figure 5a, Table 4). For all sites combined, TN and TP were both significant in predicting SCHL (*R*^2^ = 0.21 and 0.32, *p* < 0.001) (Figure 5c and 5d). Because many studies have found significant positive correlations of conductivity with nutrient concentrations (Biggs, 1995; Carpenter and Waite, 2000), TN and TP were substituted with conductivity. However, this did not improve model prediction for the three biotic variables (*R*^2^ = 0.02-0.09).

**TABLE 4:** Bivariate Regression Models for Concentrations of Periphyton Biomass (RCHL), Seston Biomass (SCHL), and Aquatic Macrophytes and/or Macroalgae (AQM) as a Function of Total Nitrogen (TN) and Total Phosphorus (TP) for All Sites Combined (*n* = 70) and for the OZRK, UMIS, and USNK Study Areas, 2006-2007.

Dependent Variable	Independent Variable	Intercept	*R*^2^	Model *p*
All sites (*n* = 70)				
log(RCHL)	0.214 log(TN)	1.628	0.08	**0.018**
log(RCHL)	−0.002 log(TP)	1.598	0.00	0.987
log(SCHL)	0.474 log(TN)	0.112	0.21	**<0.001**
log(SCHL)	0.628 log(TP)	0.936	0.32	**<0.001**
log(AQM)	0.272 log(TN)	0.980	0.04	0.083
log(AQM)	0.179 log(TP)	1.197	0.02	0.288
OZRK study area (*n* = 22)				
log(RCHL)	0.788 log(TN)	−1.657	0.16	0.064
log(RCHL)	0.056 log(TP)	1.789	0.01	0.662
log(SCHL)	0.134 log(TN)	−0.204	0.04	0.352
log(SCHL)	0.229 log(TP)	0.165	0.10	0.149
log(AQM)	0.131 log(TN)	0.802	0.01	0.641
log(AQM)	0.589 log(TP)	1.820	0.18	0.052
UMIS study area (*n* = 18)				
log(RCHL)	0.294 log(TN)	1.489	0.17	0.089
log(RCHL)	0.364 log(TP)	1.930	0.14	0.131
log(SCHL)	0.900 log(TN)	0.191	0.39	**0.005**
log(SCHL)	1.111 log(TP)	1.535	0.32	**0.015**
log(AQM)	−0.592 log(TN)	1.194	0.13	0.135
log(AQM)	−1.144 log(TP)	−0.101	0.26	**0.030**
USNK study area (*n* = 30)				
log(RCHL)	0.421 log(TN)	1.650	0.14	**0.043**
log(RCHL)	0.054 log(TP)	1.631	0.00	0.845
log(SCHL)	0.156 log(TN)	0.076	0.02	0.423
log(SCHL)	0.493 log(TP)	0.719	0.14	**0.043**
log(AQM)	0.883 log(TN)	1.221	0.34	**<0.001**
log(AQM)	−0.037 log(TP)	0.974	0.00	0.921

Note: Bold denotes *p* < 0.05.

Generally, bivariate models for specific regions did not explain significantly more variation in biotic response variables than for all sites combined. There were no significant models identified for the OZRK (Table 4). The lack of a strong TP gradient among OZRK sites could be a partial explanation for this. Significant models were identified for TN and TP in predicting SCHL (*R*^2^ = 0.39, *p* = 0.005 and *R*^2^ = 0.32, *p* = 0.015) for UMIS. TP was also identified as a significant predictor of AQM in the UMIS (*R*^2^ = 0.26, *p* = 0.030). However, this was an anomalous relation compared to other significant models, where TP concentration was inversely related to AQM (Figure 5f, Table 4). In the USNK, TN was significant in predicting AQM (*R*^2^ = 0.34, *p* < 0.001) and RCHL (*R*^2^ = 0.14, *p* < 0.043). A significant model also identified TP as important in predicting SCHL (*R*^2^ = 0.14, *p* < 0.043).

Munn *et al.* (2010) found an improvement in nutrients and algal biomass relations for open canopy sites vs. shaded sites. However, bivariate regression model results from this study used only sites with <50% canopy (*n* = 48) and generally did not improve model performance (data not shown). There were no significant models found for RCHL or AQM and nutrients. However, models for SCHL and nutrients improved slightly for TN (*R*^2^ = 0.33, *p* < 0.001) and TP (*R*^2^ = 0.41, *p* < 0.001) (see Table 4 for comparisons). In addition, there were no significant differences between means in RCHL, SCHL, and AQM for open (<50%) vs. closed (>50%) canopy sites.

### Models for the Eutrophic Index

**FIGURE 6:**
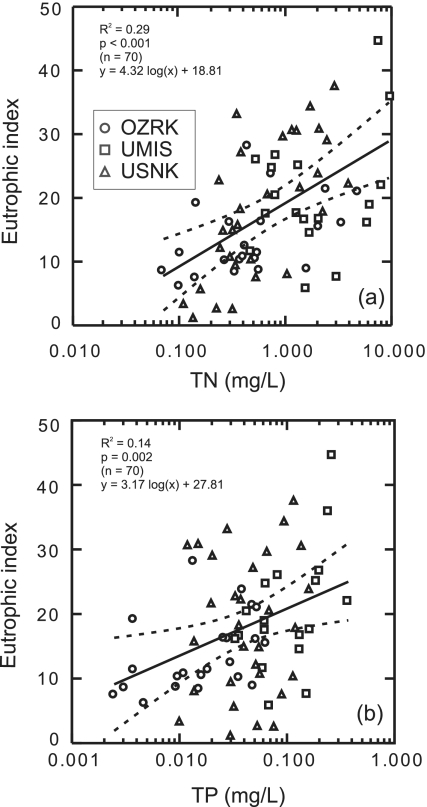
Bivariate Plots of Eutrophic Index Scores and (a) Total Nitrogen (TN) and (b) Total Phosphorus (TP) for All Study Sites. The lines indicate the best fit linear regression for all study sites. Dashed lines are the 95% confidence interval for the regression line.

**FIGURE 7:**
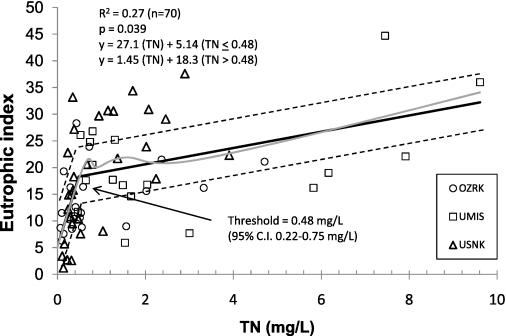
Eutrophic Index Scores as a Function of Total Nitrogen (TN) Concentrations for All Study Sites (*n* = 70). Piecewise regression line with identified breakpoint threshold for TN of 0.48 mg/l. Dashed lines are the 95% confidence interval for the regression line. The gray line represents a nonparemetric LOWESS (Locally Weighted Scatterplot Smoother) fit of the data (Helsel and Hirsch, 2002).

The majority of variance in the EI was explained by either TN (*R*^2^ = 0.29, *p* < 0.001) or TP (*R*^2^ = 0.14, *p* = 0.002) (Figures 6a and 6b), although a significant piecewise regression (*p* = 0.039) was found between TN and EI scores (Figure 7) with a breakpoint (threshold) at 0.48 mg/l (95% confidence interval of 0.22-0.75 mg/l). This threshold was the point where EI scores became less responsive to increasing TN concentrations. A nonparametric LOWESS smooth also identified a similar TN threshold. A piecewise regression and breakpoint for TP and EI scores was not significant (*p* > 0.05).

The final multivariate model for EI was a function of TN, canopy shading, median depth, and daily change in stage (adjusted *R*^2^ = 0.50, *p* < 0.001) (Table 5) with a residual standard error of 6.6 compared to a median observed score of 16. The EI scores ranged from 1.2 to 44.7 for all study sites (Table 2). Analysis of covariance using membership in each region as a binary variable did not justify a separate regression analysis for each region. Median depth appeared to be a surrogate for stream size as it was significantly correlated with discharge (*r* = 0.72, *p* < 0.05) (Table 3).

**TABLE 5:** Multiple Regression Models for Predicting Concentrations of Periphyton Biomass (RCHL), Seston Biomass (SCHL), Aquatic Macrophytes and/or Macroalgae (AQM), and Eutrophic Index (EI) as a Function of Chemical, Physical, and Biological Variables for All Sites, *n* = 70.

Dependent Variable	Predictor Variable and Coefficient	Intercept	Adjusted *R*^2^	*p-*Value
RCHL	19.04 log(TN)	56.26	0.05	0.030
SCHL	6.55 log(TP) + −2.93 log(SUBSTR) + −0.08(CAN) + −23.86(DEPTH) + 6.22 log(COND) + −3.29 log(TURB)	8.47	0.47	<0.001
AQM	122.03(DEPTH)	−1.05	0.28	<0.001
EI	10.41 log(TN) + −0.18(CAN) + 28.02(DEPTH) + 151.20(DSTAGE)	18.75	0.50	<0.001

Notes: Predictor variables listed were significant (*p* < 0.05), with most important variables listed first. See Table 2 for variable definitions.

Multivariate analysis of the individual components of the EI identified TN and median depth as important predictors for RCL and AQM, respectively (Table 5). Several nutrient and physical parameters were important in predicting SCHL concentrations, specifically increasing with TP and conductivity and decreasing with median substrate size, percent canopy, median depth, and turbidity.

## Discussion

### Physical and Chemical Characteristics

Evaluating nutrient-biotic responses in agricultural streams can be challenging because agricultural streams are known to contain some of the highest concentrations of nutrients from fertilizer and manure applications and commonly have altered riparian and instream habitats that can influence nutrient uptake and algal growth (Watzin and McIntosh, 1999; Mueller and Spahr, 2006). This can complicate nutrient gradient studies because of difficulty with locating sites with low nutrient concentrations. This study captured a strong TN gradient (0.07 to 9.6 mg/l) with 43% of sites having average concentrations below concentrations (0.52 mg/l) associated with excessive algal growth (Dodds *et al.*, 2006). Evaluating these low level nutrient conditions is important to determine if assessment methods are sensitive enough to detect biotic responses in areas that are relatively undisturbed.

The TN threshold of 0.48 mg/l using the EI scores is in close agreement with thresholds suggested by Dodds *et al.* (2006) and Stevenson *et al.* (2006) (0.52 and 0.40 mg/l, respectively) and is within the range of USEPA ecoregional TN criteria (0.31 to 0.54 mg/l) for all regions. Our identified threshold also agrees closely with the 75th percentile of TN concentrations (0.50 mg/l) measured in undeveloped watersheds across the U.S. (Clark *et al.*, 2000). A TP threshold was not identified, even though about 36% of the sites sampled had average TP concentrations below ca 0.03 mg/l reported to promote undesirable levels of algal growth (Dodds *et al.*, 2006).

Conductivity has been shown to be an indicator of water quality conditions, especially nutrient enrichment (Munn *et al.*, 2002). Many studies have reported significant positive correlations of conductivity with nutrient concentrations, particularly in relation to agriculture (Biggs, 1995; Carpenter and Waite, 2000). We also found a significant positive relation between conductivity and nutrients. However, bivariate model performance did not improve when nutrients were substituted with conductivity.

Water temperature was not important in our models, even though other studies have found a relation with plant growth (Munn *et al.*, 1989; Francoeur *et al.*, 1999). The USNK sites had the lowest mean water temperature, primarily due to higher overall elevation, but plant growth was unaffected, as there was no significant difference among regions in RCHL and AQM.

### Biological Measures of Eutrophication

We employed EI as a comprehensive measure of eutrophication using the three different forms of biotic responses to nutrients. To our knowledge, this approach has not been attempted. Our assessment demonstrates the need to evaluate eutrophication using more than one biotic response measure. Collectively, one or more eutrophic boundaries for RCHL (>70 mg/l), SCHL (>30 μg/l), and AQM (>40%) were exceeded at 46% of sites sampled, whereas exceedances based on any individual biotic measures were only 3-26%. The RCHL biomass sampled in this study represented a gradient ranging from 2.4 to 174 mg/m^2^ with about 26% of these sites considered highly enriched based on Dodds *et al.* (1998). There is no one standard RCHL biomass considered excessive or nuisance level; Dodds *et al.*’s (1998) review of the literature found excessive periphyton chlorophyll *a* ranged from 50 to 200 mg/l. Using their recommendation of 150 mg/l, there would be only 3 (4%) of our sample sites exceeding this amount, whereas our visual assessment of percent AQM coverage approaching or exceeding 40% of the reach would indicate that 15 (21%) sample sites would have excessive plant growth (Suplee *et al.*, 2009). The algal response variables RCHL and SCHL have commonly been used to evaluate nutrient enrichment, whereas AQM has received much less attention in monitoring programs. However, in many cases, RCHL or SCHL samples would not constitute eutrophication for these same sites. Indeed, high levels of both RCHL and AQM were not found in any streams (Figure 4).

The nutrient-rich agricultural streams we examined had relatively low SCHL concentrations (<10 μg/l) at 93% of all sites. Only four sites in the UMIS had higher concentrations approaching or exceeding the 30 μg/l concentration considered to be highly enriched according to Dodds *et al.* (1998). Figueroa-Nieves *et al.* (2006) found similar low concentrations (2-20 μg/l) in agricultural streams of Illinois. These findings suggest that measures of SCHL may not always be an appropriate measure of eutrophication in small agricultural streams. According to Allan (1995), the primary source of seston in fast-flowing streams is the sloughing of attached algae. However, in sluggish lowland streams or rivers where water mass has a longer residence time, conditions may be suitable for true plankton to colonize and reproduce. For example, in the tributaries for the Snake River sampled in USNK, the maximum sestonic chlorophyll *a* concentrations was 6 μg/l, whereas, in the free-flowing sections of the main-stem Snake River, concentrations approaching 100 μg/l have been reported (Myers *et al.*, 2003).

Macroinvertebrates have been shown to control RCHL, especially when hydrologic disturbance is lacking (Jacoby, 1987; Stevenson *et al.*, 2006). They found invertebrate biomass of 1-3 g/m^2^ to be sufficient to reduce periphytic algae. In addition, Sumner and McIntire (1982) found that in a laboratory stream, snails can reduce periphyton by as much as 30%. The relatively high biomass of invertebrate scrapers found at many of our study sites, many of which contained large numbers of snails, would suggest a lack of hydrologic disturbance and potential herbivory control on RCHL. However, invertebrate scrapers were not a significant explanatory variable for RCHL at our sites.

### Nutrient-Habitat-Biotic Response Models

Evaluating antecedent conditions prior to sampling validated using an index period approach that minimizes streamflow disturbance on plant growth. Continuous-stage data collected 30 days prior to sampling confirmed that high disturbances during summer index period were rare and DIMSHEAR did not exceed 0.045, which represents a threshold for bed material entrainment. High-flow disturbance was not a factor influencing biotic measures of stream eutrophication, and allowed analysis of other environmental factors on algal growth.

Numerous lotic studies have reported that nutrients are important for algal growth, but the development of nutrient regression models has not been particularly successful. Biotic measures of stream eutrophication including RCHL, SCHL, and AQM growth were not strongly related to concentrations of TN or TP; *R*^2^ values ranged from 0.02 to 0.32 for all sites combined. These results contrast with Chetelat *et al.* (1999) who reported that nutrients can become a dominant variable during stable, low-flow conditions. Our predictive models for SCHL had the highest overall *R*^2^ values, which may be partially due to autocorrelation where sestonic algal samples contain both chlorophyll *a* and nutrients (van Nieuwenhuyse and Jones, 1996; Dodds *et al.*, 2002).

Our model results to predict RCHL biomass were low (*R*^2^ = 0.0 to 0.08), but similar to those determined in other agricultural streams (*R*^2^ = 0.03) by Munn *et al.* (2010). Other studies have found stronger relations with nutrients and RCHL biomass with *R*^2^ ranging from about 0.11 to 0.60 (Lohman *et al.*, 1992; Chetelat *et al.*, 1999; Dodds *et al.*, 2006). Munn *et al.* (2010) found a negative relation between nutrients and RCHL in agricultural streams with open canopy, suggesting that periphyton can reduce TN and TP concentrations. However, performance of our model did not improve when open vs. closed canopy sites were analyzed separately.

One counterintuitive result is that model performance generally did not improve when applied to individual regions. This is contrary to Stevenson *et al.* (2006) who found correlations between measures of algal biomass and nutrients were higher when observations were constrained to a specific region vs. when data were combined. Perhaps this difference may have to do with our site selection that targeted agricultural watersheds where streams may have similar conditions including elevated nutrients and altered habitat. For example, we found no statistical differences among regions in some key environmental variables including conductivity, turbidity, and percent canopy all of which have been shown to be related to algal biomass in other streams (Biggs, 1995; Figueroa-Nieves *et al.*, 2006; Munn *et al.*, 2010). Highest mean concentrations of TN and TP were observed in the UMIS. However, these sites had the lowest mean RCHL compared with other regions, which may be due to generally finer substrate in UMIS (Biggs and Shand, 1987).

Our best model integrated all three biotic measures into a comprehensive index of stream eutrophication. EI was related to nutrient concentrations (TN and TP) (Figure 6), but nutrient concentrations alone provide a poor explanation of aquatic plant growth responses in these streams. Adding environmental factors that related to light availability (percent canopy), streamflow conditions during sampling (median depth), and recent streamflow variability (daily change in stage) improve the model estimates of eutrophic conditions accounting for 50% of the variance in EI scores. Aquatic plant growth increased with TN and decreased with percent canopy as expected, but it increased with median depth and daily change in stage. Depth appears to be a surrogate for stream size, whereas daily change in stage is a measure of water motion that has been shown to enhance nutrient uptake and availability up to a point when the force of moving water is too great and plant growth is sheared from the substrate (Horner *et al.*, 1990; Chambers *et al.*, 1991).

The findings of this study suggest that trophic-state classification of streams is more appropriately based on areal plant growth than on nutrient concentrations of TN and TP. This would be especially true for streams that contain an abundance of macrophytes. Future studies may want to consider separate visual coverage of macrophyte and macroalgae because rooted macrophytes generally uptake nutrients from bed sediments through their roots whereas macroalgae uptake nutrients from the water column (Chambers *et al.*, 1999). Each type of response (RCHL, SCHL, and AQM) may have distinct controls, but overall these responses are regulated by nutrients, light, and streamflow. Nutrient criteria may be justified as a management tool, but do not serve as a precise basis for classifying the eutrophic status of streams.

## Supplemental material

**Appendix S1.** Stream name, study unit, USGS site number, site location, sample year, and stage device used (G=USGS gage or S=stage recorder), *n*=70.

Please note: Neither AWRA nor Wiley-Blackwell is responsible for the content or functionality of any supporting materials supplied by the authors. Any queries (other than missing material) should be directed to the corresponding author for the article.
