# Supplementary material for: Influence of Environmental Factors on Biotic Responses to Nutrient Enrichment in Agricultural Streams
Source: J Am Water Resour Assoc. 2010 Jun;46(3):498–513. doi: 10.1111/j.1752-1688.2010.00430.x (PMC3307628; doi:10.1111/j.1752-1688.2010.00430.x)
Supplement: Supplementary file 1 [file jawr0046-0498-SD1.pdf]

Appendix 1. Stream name, study unit, USGS site number, site location, sample year,

and stage device used (G = USGS gage or S = stage recorder), n = 70.

Stream	Study Unit	State	Station Number	Latitude	Longitude	Year	Gage/Stage
Woods Fork	OZRK	MO	06927590	37.24528	-92.56778	2006	S
West Piney Creek	OZRK	MO	06928800	37.28111	-92.10472	2006	S
Meramec River	OZRK	MO	07010335	37.68884	-91.42514	2006	S
Long Creek	OZRK	AR	07053203	36.36408	-93.27052	2006	S
Yocum Creek	OZRK	AR	07053250	36.45432	-93.35600	2006	G
Calf Creek	OZRK	AR	07055893	35.96694	-92.77572	2006	S
Water Creek	OZRK	AR	07056695	36.04980	-92.57627	2006	S
Big Creek	OZRK	AR	07057100	35.97869	-92.48155	2006	S
Bennetts River	OZRK	AR	07058970	36.42780	-92.08278	2006	G
Roasting Ear Creek	OZRK	AR	07060661	35.92213	-92.23108	2006	S
North Sylamore Creek	OZRK	AR	07060710	35.99174	-92.21404	2006	G
Poke Bayou Creek	OZRK	AR	07060890	35.95427	-91.69863	2006	S
Sullivan Creek	OZRK	AR	07060894	35.88755	-91.64162	2006	S
Barren Fork	OZRK	MO	07064780	37.34622	-91.39089	2006	S
Big Creek	OZRK	MO	07065040	37.31310	-91.31680	2006	S
Mahans Creek	OZRK	MO	07065950	37.14727	-91.37847	2006	S
South Fork Spring River	OZRK	AR	07069267	36.48430	-91.84670	2006	S
Piney Creek	OZRK	AR	07050228	36.26813	-93.63519	2006	S
Shoal Creek	OZRK	MO	07186670	36.77697	-94.02411	2006	S
North Indian Creek	OZRK	MO	07188855	36.81118	-94.21021	2006	S
Beaty Creek	OZRK	OK	07191222	36.36560	-94.72750	2006	G

Contnued-Appendix 1

Little Osage Creek	OZRK	AR	07194947	36.25369	-94.27021	2006	S
Mississippi River	UMIS	MN	05200170	47.32556	-95.22500	2007	S
Shell River	UMIS	MN	05243200	46.81417	-95.12139	2007	S
Swan River	UMIS	MN	05265698	45.93056	-94.43083	2007	S
North Two Rivers	UMIS	MN	05267185	45.82278	-94.47250	2007	S
Spunk Creek	UMIS	MN	05267578	45.76083	-94.33667	2007	S
Skunk River	UMIS	MN	05267930	45.92722	-94.17750	2007	S
Little Rock Creek	UMIS	MN	05268700	45.76333	-94.20417	2007	S
Middle Fork Crow River	UMIS	MN	05278020	45.22694	-94.72639	2007	S
Cedar Creek	UMIS	MN	05286297	45.33278	-93.30083	2007	S
Elm Creek	UMIS	MN	05287890	45.16333	-93.43639	2007	G
Chetomba Creek	UMIS	MN	05314510	44.84000	-95.23889	2007	G
South Branch Rush River	UMIS	MN	05326189	44.46528	-94.15000	2007	G
Wood River	UMIS	WI	05338955	45.78528	-92.63111	2007	S
Trade River	UMIS	WI	05340280	45.68000	-92.64583	2007	S
Apple River	UMIS	WI	05340962	45.45667	-92.30500	2007	S
Willow River	UMIS	WI	05341685	45.16556	-92.38000	2007	S
Valley Branch	UMIS	MN	05341763	44.91583	-92.79000	2007	S
West Fork Beaver Creek	UMIS	MN	531656290	44.69028	-95.03417	2007	G
Willow Creek	USNK	ID	13057940	43.44330	-111.72913	2007	G
Blackfoot River	USNK	ID	13063000	42.81572	-111.51144	2007	G
Portneuf River	USNK	ID	13073000	42.62431	-112.08869	2007	G

Continued-Appendix 1

Marsh Creek	USNK	ID	13075000	42.62989	-112.22523	2007	G
Mink Creek	USNK	ID	13075320	42.75153	-112.39494	2007	S
Raft River	USNK	ID	13078000	42.06460	-113.45103	2007	G
Marsh Creek	USNK	ID	13082300	42.45251	-113.52216	2007	S
Goose Creek	USNK	ID	13082500	42.12755	-113.93468	2007	G
Trapper Creek	USNK	ID	13083000	42.15712	-113.99764	2007	G
Big Cottonwood Creek	USNK	ID	13088510	42.29371	-114.02294	2007	S
Devils Washbowl Spring[1]	USNK	ID	13089500	42.58930	-114.34719	2007	G
Blue Lakes Spring[1]	USNK	ID	13090999	42.61473	-114.46922	2007	G
Rock Creek	USNK	ID	13091995	42.32218	-114.26844	2007	S
North Cottonwood Creek	USNK	ID	13092300	42.32972	-114.42592	2007	S
Rock Creek	USNK	ID	13092747	42.56058	-114.49519	2007	G
Cedar Draw	USNK	ID	13093478	42.56724	-114.62934	2007	S
Mud Creek	USNK	ID	13094680	42.62634	-114.80542	2007	S
Briggs Spring1	USNK	ID	13095175	42.67394	-114.80871	2007	G
Blind Canyon Spring[1]	USNK	ID	13095400	42.70333	-114.82222	2007	S
Box Canyon Springs[1]	USNK	ID	13095500	42.70750	-114.81770	2007	G
Salmon Falls Creek	USNK	NV	13103510	41.94313	-114.68631	2007	G
Shoshone Creek	USNK	NV	13104900	41.94645	-114.68569	2007	S
Salmon Falls Creek	USNK	ID	13107200	42.45323	-114.86391	2007	S
Billingsley Creek[1]	USNK	ID	13134640	42.79241	-114.86540	2007	S
Big Wood River	USNK	ID	13140800	43.32654	-114.32181	2007	G

Continued-Appendix 1

Willow Creek	USNK	ID	13140900	43.33030	-114.29107	2007	S
Rock Creek	USNK	ID	13141070	43.36419	-114.39810	2007	S
Camas Creek	USNK	ID	13141500	43.33276	-114.54223	2007	G
Stalker Creek[1]	USNK	ID	13150200	43.31123	-114.18023	2007	S
Clover Creek	USNK	ID	13154400	43.00139	-115.18138	2007	S

[1] streamflow predominantly from nearby spring sources
